# Material Composition Design for Long-Term Stability of Solid Oxide Fuel Cell Based on Creep Damage and Failure Probability

**DOI:** 10.3390/ma19040733

**Published:** 2026-02-13

**Authors:** Yu Wang, Ming Song

**Affiliations:** 1 College of Transportation, Shandong University of Science and Technology, Qingdao 266590, China; 2 Department of Engineering Mechanics, College of Pipeline and Civil Engineering, China University of Petroleum (East China), Qingdao 266555, China; songmingx@gmail.com

**Keywords:** SOFC, creep damage, electrode, material composition, failure probability

## Abstract

Electrode degradation represents a primary factor contributing to the performance decay. The composition design of electrode materials directly determines the long-term stability of solid oxide fuel cell (SOFC) under high-temperature service conditions. This paper focuses on the effect of anode and cathode material compositions on the creep damage and failure probability of SOFCs after 50,000 h creep. The results reveal an optimal Ni content range for mechanical integrity. Specifically, increasing the Ni volume fraction from 30% to 50% results in a reduction in the creep damage. In contrast, extending the increase to 60–70% causes a general reversal of this trend, with the creep damage showing an overall increase. The paper concludes that the Ni volume fraction of 50–60% is appropriate to maintain the long-term operation of SOFC. The La_0.8_Sr_0.2_MnO_3_ (LSM) volume fraction with higher electrochemical efficiency can be selected for cathode manufacturing. This study provides a reference for developing long-life SOFC electrode materials.

## 1. Introduction

A solid oxide fuel cell (SOFC), as a clean and efficient energy conversion device, is one of the major strategic approaches to achieving carbon neutrality [1,2,3]. The operating principle is based on electrochemical reactions, directly converting chemical energy into electrical energy [4]. Within solid oxide fuel cells, the anode serves as the site where fuel oxidation reactions (such as H_2_, CH_4_, etc.) occur in SOFCs [5]. Currently, nickel–zirconium stabilized zirconia (Ni-YSZ) composite anodes represent the most extensively employed anode material, owing to their advantages of lower production cost and outstanding electrical conductivity [6]. The structural stability of all-oxide anodes primarily depends on the stability of their crystal structure, ion/electron conductivity properties, and resistance to reduction [7,8]. These properties are all closely related to the composition design of the material. The cathode serves as the site for the oxygen reduction reaction in SOFCs [9]. Currently, perovskite-type oxides (such as La_0.8_Sr_0.2_MnO_3_ (LSM) and Ba_0.5_Sr_0.5_Co_0.8_Fe_0.2_O_3−δ_ (BSCF)) are the primary cathode materials for SOFCs [10]. The long-term structural stability of cathode materials directly influences reaction kinetics. Their degradation mechanisms primarily include grain growth, surface element segregation and volatilization, interfacial reactions, and oxygen vacancy ordering [11,12]. These degradation behaviors are all closely related to the composition design of the materials. Therefore, the composition design of the electrodes is of great importance.

Currently, the composition design of electrode materials is primarily based on electrochemical efficiency. Besides the above-mentioned problems, mechanical damage can also be caused by the mismatched component design. SOFCs typically operate at temperatures as high as 600–1000 °C. Currently, there is also significant research focused on developing proton-conducting SOFCs that can operate at intermediate temperatures (400–600 °C) [13,14,15]. Despite these advancements, the mismatched thermal expansion coefficients between layers can still lead to the generation of residual stresses and thermal stresses, leading to creep damage, fatigue damage, and plastic deformation. These problems lead to a decrease in SOFC output power and a shortened lifespan, which seriously restricts their commercialization process [16,17,18].

For composite materials like the anode and cathode, their material properties are closely related to the proportion of components. The elastic modulus and coefficient of thermal expansion (CTE) are close to the material properties of the component with a higher content proportion. Therefore, changing the component proportion of the composite electrode in the structure will affect the residual stress and thermal stress of the structure. Increasing the Ni content in the anode improves electronic conductivity at the expense of a heightened CTE mismatch within the positive electrode–electrolyte–negative electrode (PEN) assembly [19]. Fang et al. [19] performed numerical simulations to study the effect of anode Ni content on the mechanical failure probability of planar SOFC. The failure probability of anode increases with the increase in Ni content from 30% to 70%. Compared to thickness and Young’s modulus, the CTE exerts a more dominant influence on residual stress, as reported by Tanaka et al. [20]. Their study demonstrated that increasing the CTE of the anode substrate layer effectively mitigates tensile residual stress within the diffusion barrier interlayer. Conversely, a reduction in the CTE of the interlayer itself also contributes to lower residual stress levels. Mansur et al. [21] attributed the significant performance limitations of the cell to a substantial CTE mismatch between the cathode and electrolyte. This incompatibility generated considerable mechanical stress, increased interfacial resistance, and promoted structural degradation, consequently leading to a substantially reduced power output. They concluded that CTE matching is a paramount design criterion, dictating SOFC performance and durability.

Different material parameters lead to distinct stress distributions, which is a direct factor causing creep and damage. Ma et al. [22] established a strain-based creep damage and failure probability model to perform a time-dependent mechanical performance analysis of SOFC. The authors emphasized the necessity of safety inspections beyond 55,000 h to maintain structural integrity. Jiang et al. [23] found that the bonded compliant seal with Ag–4%CuO and BNi-2 filler metal demonstrates better creep strength than the rigid seal with a glass ceramic in SOFC. In addition, the change in material composition will also lead to the change in creep performance of the material, which influences the long-term stability of SOFC under high-temperature service conditions. We [24] found that by increasing the creep parameter of the frame, the creep and damage in the cell and sealant are decreased. Zhang et al. [25] found that the maximum creep damage, damage area and failure probability of the sealant layer all increase by increasing the creep strength coefficient of the sealant layer.

In conclusion, there are significant differences in structural stability among electrodes with different component designs during long-term high-temperature operation. To meet the reliability requirements of SOFCs, designers usually select appropriate materials based on electrical performance. However, there is an insufficient design basis in current SOFC engineering applications, especially the lack of relevant experience in terms of long-term reliability. Therefore, it is necessary to study the effect of anode and cathode material composition on the creep life of SOFCs, so as to provide a foundation for optimized design. Based on the SOFC ductile exhaustion creep damage–time-dependent failure probability model, this paper conducts a study on the material composition affecting the creep failure of SOFCs, thereby providing a basis for the optimized composition design and development of high-stability electrode materials.

## 2. Method and Modeling

### 2.1. Computational Model

**Figure 1:**
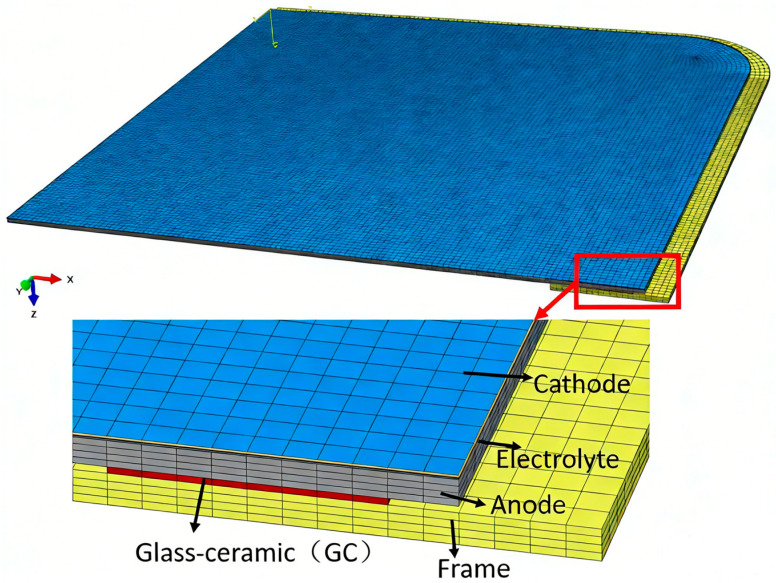
FEM model of SOFC.

The finite element method (FEM) model employed in this study is shown in Figure 1. In the simulation process of this paper, the geometric model, number of mesh elements and nodes, and boundary conditions of the finite element model are consistent with those in the finite element simulation process in Ref. [26]. The related material parameters are listed in Table 1 [26].

### 2.2. Creep Damage Model and Failure Probability Calculation Equation

The creep constitutive equation incorporating a creep damage variable from a micro-mechanics perspective (the Wen-Tu model) [27] is used:

(1) $${\dot{\varepsilon }}_{ij}^{c}=\frac{3}{2}B\sigma_{\mathrm{eq}}^{n-1}S_{ij}{\left[1+\beta {\left(\frac{\sigma_{1}}{\sigma_{\mathrm{eq}}}\right)}^{2}\right]}^{\frac{n+1}{2}}$$

(2) $$\beta =\frac{2\rho }{n+1}+\frac{\left(2n+3\right)\rho^{2}}{n{\left(n+1\right)}^{2}}+\frac{\left(n+3\right)\rho^{3}}{9n{\left(n+1\right)}^{3}}+\frac{\left(n+3\right)\rho^{4}}{108n{\left(n+1\right)}^{4}}$$

(3) $$\rho =\frac{2\left(n+1\right)}{\pi \sqrt{1+3/n}}\omega^{3/2}$$

(4) $$\omega =\int_{0}^{t}\frac{{\dot{\varepsilon }}^{c}}{\varepsilon_{f}^{*}}$$

(5) $$\frac{\varepsilon_{f}^{*}}{\varepsilon_{f}}=\exp\left(\frac{2}{3}\left(\frac{n-0.5}{n+0.5}\right)\right)/\exp\left(2\left(\frac{n-0.5}{n+0.5}\right)\frac{\sigma_{m}}{\sigma_{\mathrm{eq}}}\right)$$

where ${\dot{\varepsilon }}_{ij}^{c}$ and ${\dot{\varepsilon }}^{c}$ refer to the creep strain tensor rate and the equivalent creep strain rate, respectively. *B* and *n* represent the material’s creep constants. *S_ij_* is the stress deviator, and $\sigma_{1}$, $\sigma_{eq}$ and $\sigma_{m}$ are the maximum principal stress, von Mises equivalent stress and the hydrostatic stress, respectively. β is a stress-dependent function, and ρ is the micro-crack damage parameter. ω is the damage variable, $\varepsilon_{f}^{*}$ and $\varepsilon_{f}$ refer to the multi-axial fracture strain at the creep stage and the uniaxial creep failure strain, respectively.

The distribution of internal defects in ceramic materials exhibits a certain degree of randomness. Therefore, the failure probability is commonly used to characterize the failure of ceramic materials. A constitutive model is utilized to compute the time-dependent failure probability $P_{f}^{c}\left(\varepsilon^{c},V_{j}\right)$ during the creep, as follows [28]:

(6) $$P_{f}^{c}\left(\varepsilon^{c},V_{j}\right)=\int_{0}^{\varepsilon^{c}}f\left(\varepsilon_{f}^{*}\right)d\varepsilon_{f}^{*}=1-\exp\left[-{\int_{V_{j}}\left(\frac{\varepsilon^{c}}{\eta }\right)}^{m}\frac{dV_{j}}{V_{0}}\right]$$

where η is the characteristic creep strain, *m* represents the Weibull modulus, and *V*_0_ and *V_j_* refer to the reference volume and the material volume, respectively. The related material parameters are listed in Table 2 [28,29].

### 2.3. Material Properties of the Composite Materials

Since the proportion of constituent materials directly determines the effective properties of the composite, the composite sphere model (CSM) [30] is applied to compute the mechanical properties of the dense anode and cathode:

(7) $$K_{com}=K_{2}+\frac{\psi_{1}}{1/\left(K_{1}-K_{2}\right)+3\psi_{2}/\left(3K_{2}-4G_{2}\right)}$$

(8) $$G_{com}=G_{2}+\frac{\psi_{1}}{1/\left(G_{1}-G_{2}\right)+6\psi_{2}\left(3K_{2}+2G_{2}\right)/\left(5G_{2}\left(3K_{2}+4G_{2}\right)\right)}$$

(9) $$K=\frac{E}{3\left(1-2\nu \right)}$$

(10) $$G=\frac{E}{2\left(1+\nu \right)}$$

where *K_com_* and *G_com_* are the bulk modulus and shear modulus of the dense composite material, respectively, while *K*_2_ (*K*_1_) and *G*_2_ (*G*_1_) are the bulk and shear moduli of the matrix (impurity) in the composite, respectively. ψ represents the volume fraction of the material. The material properties of the dense materials of the matrix and impurity in each composite material of SOFC are shown in Table 3.

In addition to the proportion of each component, the porosity of the material also has a significant impact on the Young’s modulus and shear modulus of the composite material. The relationship between Young’s modulus and shear modulus and the volume fraction of porosity can be expressed by semi-empirical formulas summarized from empirical and theoretical models. Based on the ‘composite sphere model’, the effective elastic modulus, bulk modulus, and Poisson’s ratio of the composite material can be obtained as follows [31]:

(11) $$E=E_{0}{\left(1-p\right)}^{2}/\left(1+b_{E}p\right)$$

(12) $$G=G_{0}{\left(1-p\right)}^{2}/\left(1+b_{G}p\right)$$

(13) $$\nu =0.25\left(4\nu_{0}+3p-7\nu_{0}p\right)/\left(1+2p-3\nu_{0}p\right)$$

In which $\nu_{0}$ is the Poisson’s ratio of dense material. $b_{E}=\left(2-3\nu_{0}\right)$, $b_{G}=\left(11-19\nu_{0}\right)/\left(4+4\nu_{0}\right)$. *p* is the porosity of the material.

The effective thermal expansion coefficient of the composite electrode is as follows [32]:

(14) $$\alpha_{com}=\frac{\alpha_{1}\psi_{1}K_{1}+\alpha_{2}\psi_{2}K_{2}}{\psi_{1}K_{1}+\psi_{2}K_{2}}$$

The coefficient of thermal expansion is as shown below:

(15) $$CTE_{anode}=CTE_{YSZ}+\left(CTE_{Ni}-CTE_{YSZ}\right)\psi_{Ni}\left(0.1+0.9\psi_{Ni}\right)$$

(16) $$CTE_{cathode}=CTE_{YSZ}+\left(CTE_{LSM}-CTE_{YSZ}\right)\psi_{LSM}\left(0.1+0.9\psi_{LSM}\right)$$

Assuming that the reduction in the total volume of the anode after reduction is negligible, the porosity *p* of the anode sample after reduction can be derived as a function of the initial porosity *p*_0_ before reduction [33]:

(17) $$p=p_{0}+p_{0}{\bar{m}}_{NiO}^{0}\left[\frac{1}{\rho_{NiO}}+\frac{1}{\rho_{Ni}}-\left(\frac{m_{O}}{m_{NiO}}\right)\left(\frac{1}{\rho_{Ni}}\right)\right]r$$

where $p_{0}$, $\rho_{0}$, $\rho_{Ni}$ and $\rho_{NiO}$ are the average initial porosity, initial density of the sample, density of Ni, and density of NiO, respectively. ${\bar{m}}_{NiO}^{0}$ is the initial weight fraction of NiO in the NiO-YSZ composite. *m_O_* and *m*_NiO_ represent the atomic mass numbers of oxygen and nickel oxide, respectively. *r* is the volume fraction of reduced NiO-YSZ.

## 3. Results and Discussion

### 3.1. Effect of Ni Volume Fraction

Since the elastic modulus of NiO is approximately equal to that of YSZ, the variation in the elastic modulus of NiO-YSZ with the volume fraction of NiO can be neglected. The variation curve of the elastic modulus of dense Ni-YSZ with the volume fraction of Ni can be obtained. Then, based on Equation (5), the dependence of the ‘true’ elastic modulus on the Ni volume fraction with a certain porosity can be calculated. The influence of $\psi_{Ni}$ within the range of 0.3 to 0.7 is computed.

**Figure 2:**
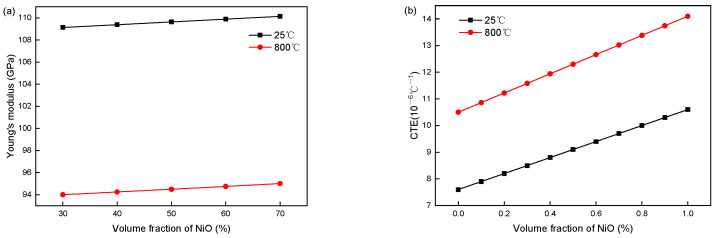
(**a**) Elastic modulus and (**b**) thermal expansion coefficient of NiO-YSZ change with NiO volume fraction at 25 °C and 800 °C.

**Figure 3:**
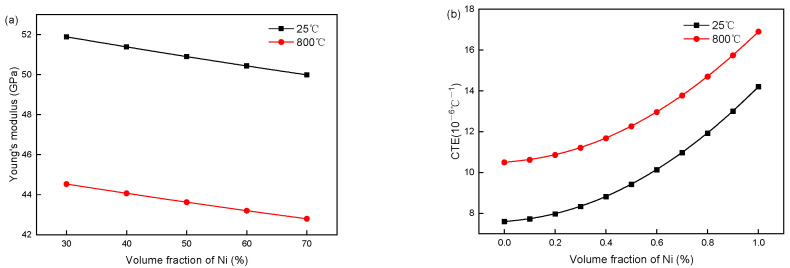
(**a**) Elastic modulus and (**b**) thermal expansion coefficient of Ni-YSZ change with Ni volume fraction at 25 °C and 800 °C.

At an initial NiO weight fraction of 0.55 (*ρ*_NiO_ = 8.88 g/cm^3^, *ρ*_Ni_ = 6.67 g/cm^3^, and *p*_0_ = 0.23), the porosity of the composite reaches 0.4 after reduction, which results in the material parameters of Ni/NiO-YSZ being closely dependent on the Ni/NiO volume fraction (Figure 2 and Figure 3). For NiO-YSZ composites, the elastic modulus and CTE both exhibit a slight increasing trend with rising NiO volume fraction: the elastic modulus increases marginally from 109.13 GPa to 110.14 GPa at 25 °C and from 94 GPa to 95 GPa at 800 °C, while the CTE rises from 8.5 × 10^−6^/°C to 9.7 × 10^−6^/°C at 25 °C and from 11.58 × 10^−6^/°C to 13.02 × 10^−6^/°C at 800 °C. In contrast, Ni-YSZ composites show an opposite variation in elastic modulus with increasing Ni volume fraction (a decrease from 51.89 GPa to 49.99 GPa at 25 °C and 44.53 GPa to 42.8 GPa at 800 °C), accompanied by a more pronounced increase in CTE (from 8.33 × 10^−6^/°C to 10.97 × 10^−6^/°C at 25 °C and 11.21 × 10^−6^/°C to 14.7 × 10^−6^/°C at 800 °C). These contrasting trends arise from intrinsic mechanical and thermal property differences between NiO/Ni metallic phases and YSZ ceramic. At low metallic fractions, hard, low-CTE YSZ dominates the composite’s stiffness and thermal expansion. With increasing Ni content, the soft, high-CTE metallic phase gradually prevails, lowering the elastic modulus and raising the thermal expansion. The NiO-YSZ properties change little, as NiO is mechanically stable and barely disturbs the ceramic matrix.

Since the elastic modulus of Ni/NiO is approximately equal to that of YSZ, the elastic modulus of Ni/NiO-YSZ changes very little (less than 4%) with the volume fraction of Ni/NiO and can be neglected. However, due to the significant difference in CTE between Ni/NiO metallic materials and YSZ ceramic materials at the same temperature, the CTE of Ni/NiO-YSZ anodes shows a relatively obvious increasing trend with the rise in the volume fraction of Ni/NiO. In particular, the CTE of Ni-YSZ increases by more than 30%, indicating a substantial impact on the stress distribution of SOFC. Therefore, this paper only studies the effect of the volume fraction of Ni/NiO on the thermal expansion coefficient.

**Figure 4:**
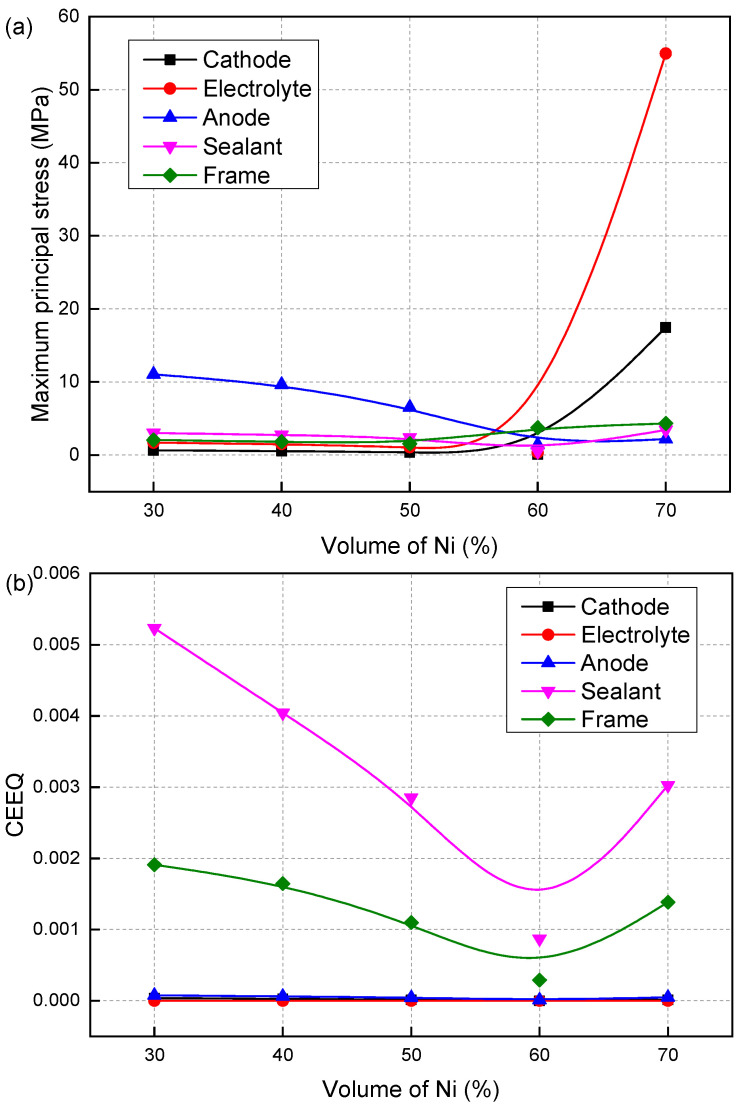

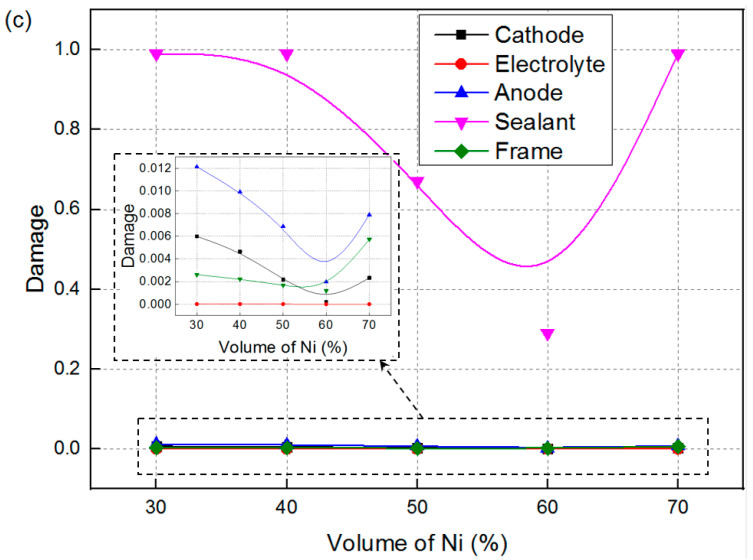
Effect of Ni volume fraction on the (**a**) maximum principal stress, (**b**) creep strain and (**c**) damage.

Figure 4 shows the effect of Ni volume fraction on the maximum principal stress, creep strain, and creep damage of SOFC. As the Ni volume fraction increases, the maximum principal stress after creep, creep strain, and creep damage gradually decrease, reaching a minimum at 60% Ni. However, as the volume fraction increases from 60% to 70%, the maximum principal stress, creep strain, and creep damage suddenly increase again. Although the maximum principal stress in the electrolyte increases significantly from 0.35 MPa to around 60 MPa, the creep damage of the electrolyte is very small (less than 0.001). This is due to the high strength of the dense electrolyte compared to the other porous components. The creep damages of the cathode, anode and metal framework are all below 0.1. However, the sealant shows far higher creep strain and damage values than other layers, due to its lower creep strength. As shown in Table 2, GC has a high stress exponent. Despite its low stress level, GC exhibits relatively large creep strain and damage. The sealant damage reaches 0.99 at 30% and 40% Ni. At 50% and 60% Ni, the damage values drop to 0.67 and 0.29, corresponding to reductions of 32.3% and 70.7%. When the Ni volume fraction is 70%, the damage value of the sealant reaches 0.99 again. The significant increases in the maximum principal stress and creep damage of the SOFC caused by the increase in Ni volume fraction from 60% to 70% are mainly due to the change in the thermal expansion coefficient of the anode material induced by the higher Ni proportion, which in turn enhances the mismatch between the various materials.

**Figure 5:**
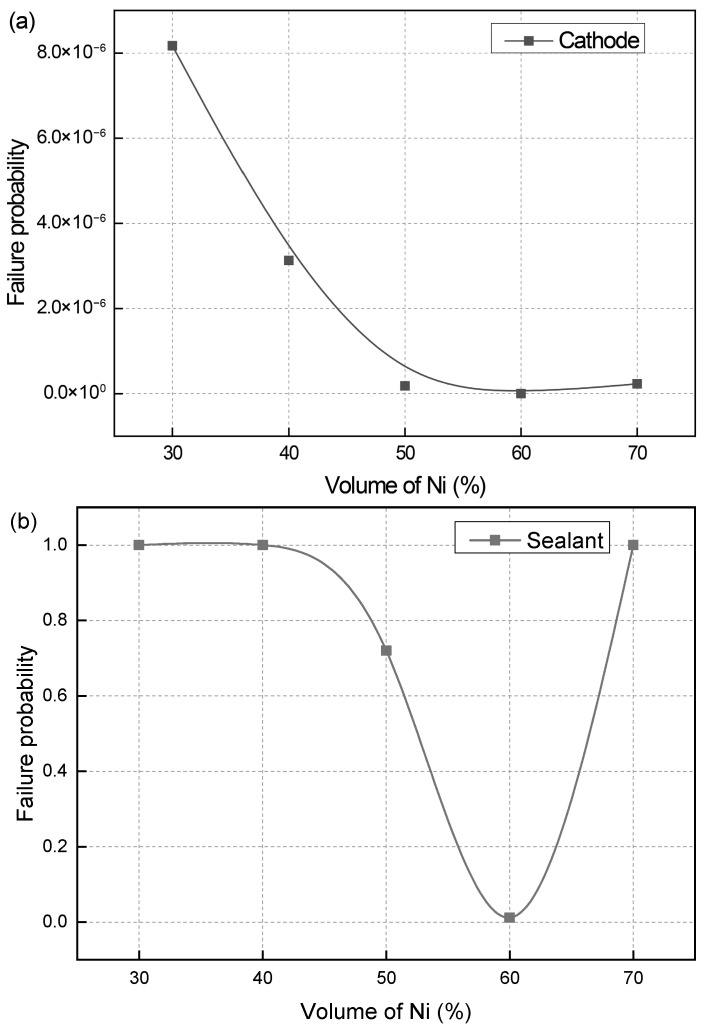
Effect of Ni volume fraction on the failure probability of (**a**) cathode and (**b**) sealant.

Figure 5 shows the effect of the Ni volume fraction on the failure probability of the cathode and sealant, with similar influencing patterns and damage characteristics. The failure probability of the electrolyte and anode remains at 0 at all Ni contents, due to negligible creep strain during operation. For the cathode, the failure probability varies with the Ni fraction, peaking at 8.172 × 10^−6^ at 30% Ni and falling to 2.973 × 10^−12^ at 60% Ni. All values stay within the safe range. In contrast, the sealant undergoes larger creep strain, leading to a markedly higher failure probability and making it the most failure-prone and critical component in the SOFC. Its failure probability reaches one at 30% and 40% Ni, indicating complete structural failure and loss of normal SOFC operational capacity. At 50% and 60% Ni, the values drop to 0.72 and 0.012, but rise back to one at 70%. These distinct failure probability trends stem from the strong correlation between the Ni volume fraction and the overall thermal–mechanical compatibility of the SOFC stack. The Ni phase’s thermal expansion behavior directly modulates the internal thermal stress distribution of the stack. Deviations in Ni volume fraction from the 50–60% range exacerbate creep strain in the sealant (a component that is highly sensitive to thermal stress), leading to a sharp rise in its failure risk. This highlights that maintaining the Ni volume fraction at approximately 50–60% is the optimal choice for ensuring the long-term stable operation of SOFCs. This range optimizes the stack’s thermal–mechanical balance and minimizes the creep-induced failure risk of key components. The influence pattern of Ni volume fraction *V_Ni_* on the failure probability of the sealant can be expressed as follows:

(18) $$P_{F}(V_{Ni})=-2.1384+0.16295V_{Ni}-0.00212V_{Ni}^{2},\;(0.4<V_{Ni}<0.6)$$

where 0.4 < *V*_Ni_ < 0.6, the probability of SOFC failure is one if *V*_Ni_ exceeds this range.

**Figure 6:**
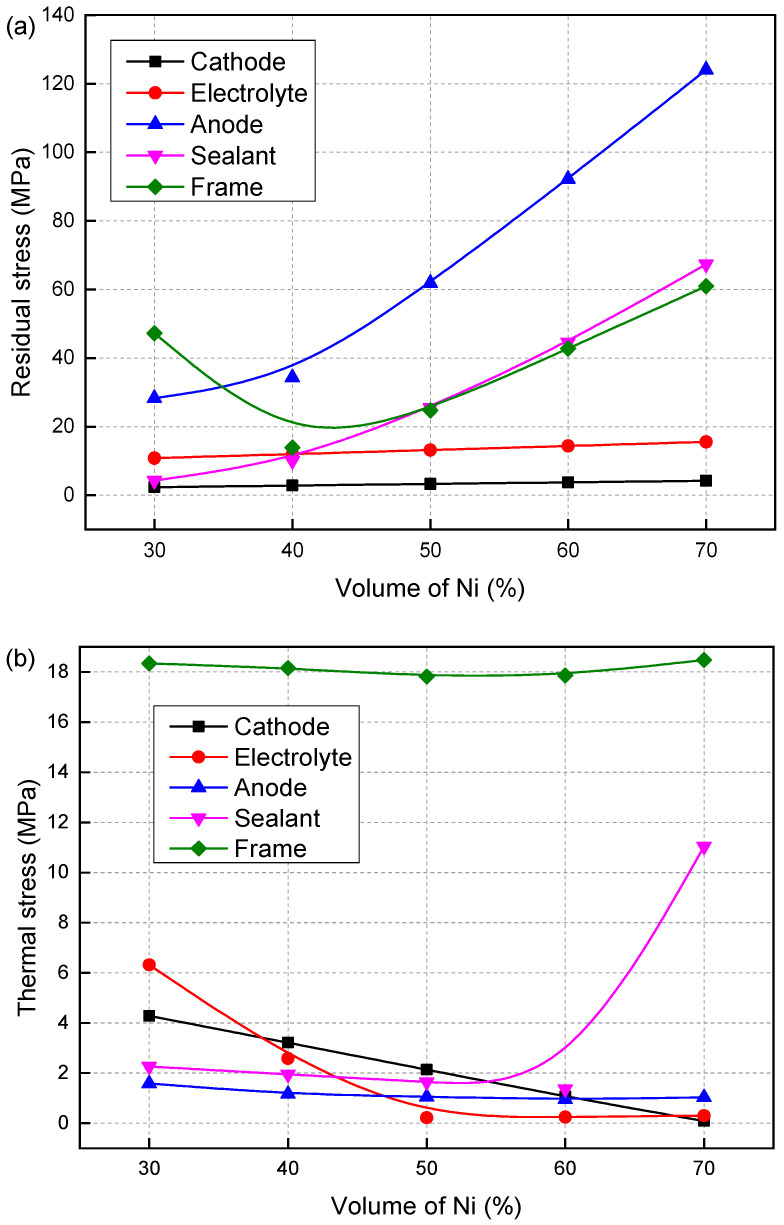
Effect of the volume fraction of Ni on the (**a**) residual stress and (**b**) thermal stress.

This paper assumes that the creep coefficients of all materials remain constant, with only the CTE of the anode varying with changes in the Ni volume fraction. The variation in the CTE directly affects the residual stress and thermal stress of the SOFC, thereby influencing its creep strain and damage. Figure 6 shows the effect of the Ni volume fraction on (a) residual stress and (b) thermal stress. Except for the metal frame, all regions of the SOFC show an increase in residual stress as the Ni volume fraction rises. At a Ni volume fraction of 60%, the residual stress of the sealant exceeds the material strength, which will cause cracking of the sealant. Therefore, the influence of residual stress cannot be ignored, and it is recommended to maintain the Ni volume fraction within the range of 50% to 60%.

Due to the rise in temperature and the influence of the thermal expansion coefficients between materials, the thermal stress values are much smaller than the residual stress values, but their distributions are completely different. The distribution of residual stress has a certain influence on the distribution of thermal stress and cannot be ignored. The initial thermal stresses of SOFCs with Ni volume fractions of 50% and 60% are not significantly different. When the Ni volume fraction is 50%, the initial thermal stresses of the electrolyte and metal framework are even smaller than when it is 60%. However, after creep, the creep strain and damage of the SOFC with a Ni volume fraction of 60% are the smallest. This is because during creep, the reduction in the anode causes stress redistribution. When the Ni volume fraction is 60%, the thermal expansion coefficients of NiO-YSZ in the oxidized state and Ni-YSZ in the reduced state are the closest. The changes in material parameters caused by the anode reduction have a smaller impact.

### 3.2. Effect of LSM Volume Fraction

**Figure 7:**
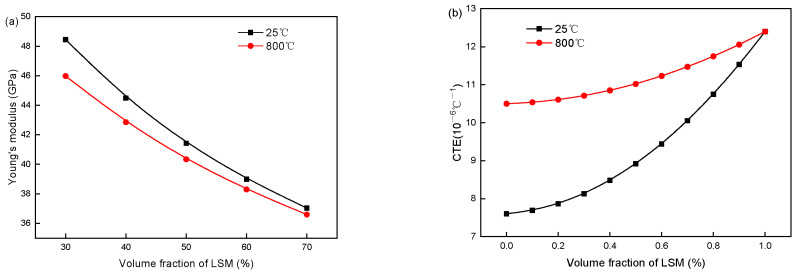
(**a**) Elastic modulus and (**b**) thermal expansion coefficient of LSM-YSZ change with LSM volume fraction at 25 °C and 800 °C.

Figure 7 depicts the evolutions of elastic modulus and CTE for LSM-YSZ composites with varying LSM volume fractions at 25 °C and 800 °C. With the LSM volume fraction increasing from 30% to 70%, the elastic modulus of LSM-YSZ decreases by approximately 23% (from 48.44 GPa to 37.04 GPa) at 25 °C and 20% (from 45.98 GPa to 36.6 GPa) at 800 °C, whereas the CTE rises by 23% (from 8.133 × 10^−6^/°C to 10.053 × 10^−6^/°C) at 25 °C and a mild 7% (from 10.71 × 10^−6^/°C to 11.47 × 10^−6^/°C) at 800 °C. These trends result from inherent property differences between LSM and YSZ (Table 3). YSZ has a higher elastic modulus, while LSM has a higher CTE. Increasing LSM weakens the stiffening effect of YSZ, leading to a gradual reduction in the overall elastic modulus. Meanwhile, the thermal expansion behavior of LSM-YSZ is increasingly dominated by LSM, resulting in a continuous rise in CTE. Notably, the elastic modulus and CTE of LSM-YSZ exhibit an inverse correlation with changing LSM content. Their combined effects on the internal stress distribution of the composite are non-intuitive and difficult to predict through qualitative analysis alone. It is necessary to calculate the actual stress distribution, conduct detailed comparisons, and select an appropriate LSM volume fraction.

**Figure 8:**
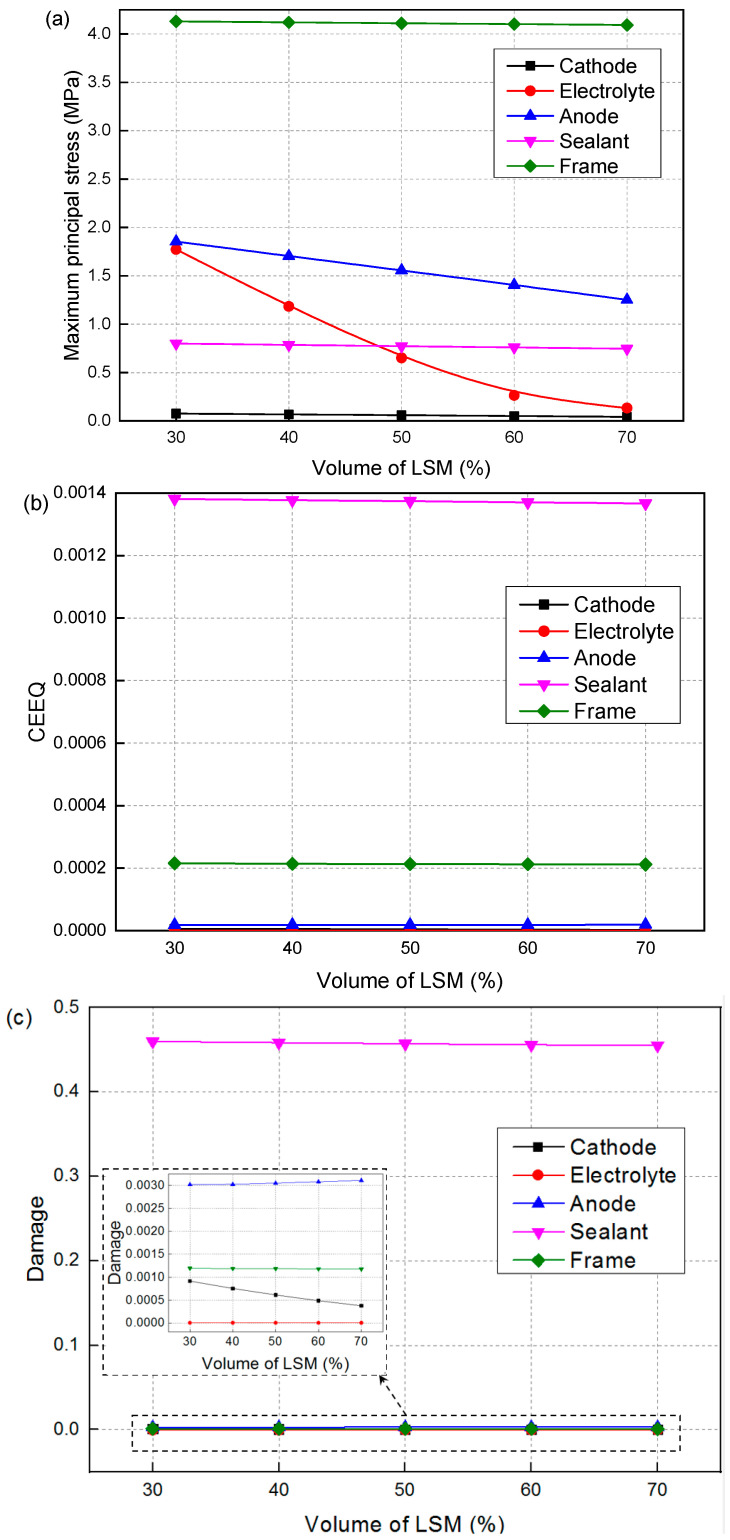
Effect of LSM volume fraction on the (**a**) maximum principal stress, (**b**) creep strain and (**c**) damage.

Figure 8 shows the effect of LSM volume fraction on the maximum principal stress, creep strain, and creep damage of SOFC. As the LSM volume fraction decreases, the maximum principal stress after creep gradually decreases. The stress of the electrolyte decreases from 1.773 to 0.134, a reduction of approximately 93%. As the LSM volume fraction decreases, the creep strain and damage of the cathode, sealant, and metal frame gradually decrease, while the creep strain and creep damage of the electrolyte and anode gradually increase. Among them, the creep strain of the cathode decreases from 5.511 × 10^−6^ to 2.258 × 10^−6^, and the creep damage decreases from 9.171 × 10^−4^ to 3.757 × 10^−4^. Although the reduction is significant (~59%), the values are small and have little impact. The sealant exhibits relatively high levels of creep strain and damage, but the changes are very small (~1%). In contrast, the other components demonstrate low values of both creep strain and damage, accompanied by negligible changes. It can be seen that the influence of the LSM volume fraction is not significant.

**Figure 9:**
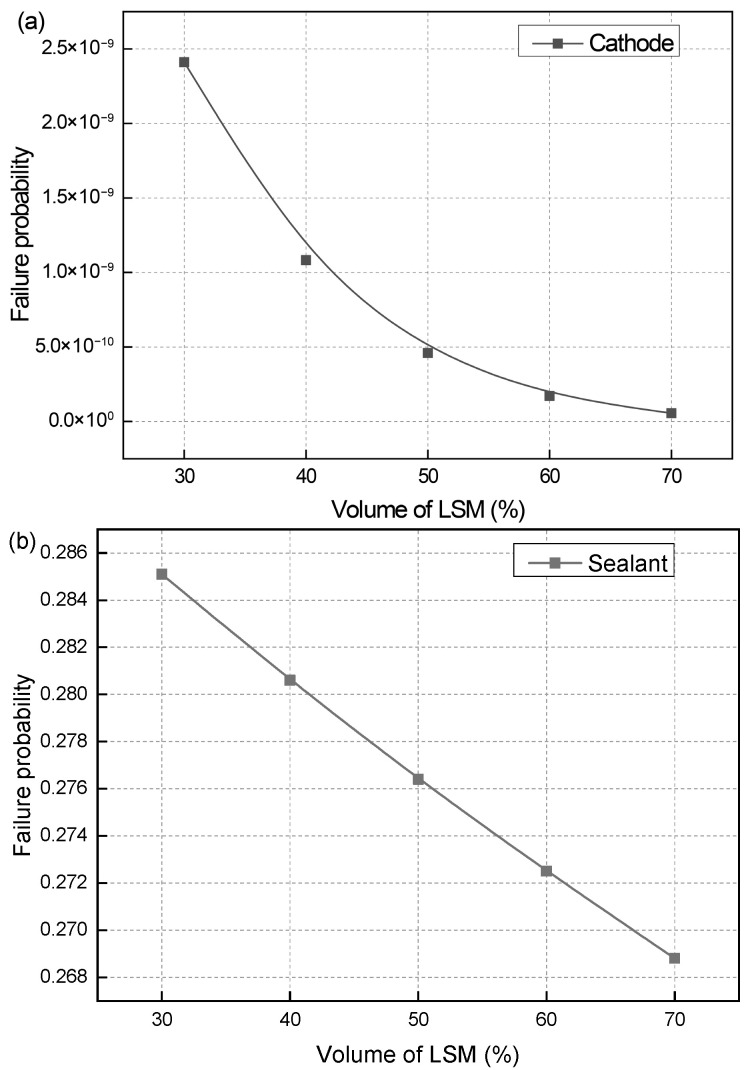
Effect of LSM volume fraction on the failure probability of (**a**) cathode and (**b**) sealant.

Figure 9 shows the effect of the LSM volume fraction on the failure probability of the cathode and sealant. Since the creep strain of the electrolyte and anode is very small, their failure probabilities are both 0 and they are not listed here. As the LSM volume fraction arises from 30% to 70%, the failure probability of the cathode gradually decreases from 2.412 × 10^−9^ to 5.518 × 10^−11^. The failure probability values are very small and all within the safe range. Due to the relatively large creep strain of the sealant, its failure probability is also correspondingly larger. When the LSM volume fraction increases from 30% to 70%, the failure probability of the sealant gradually decreases from 0.285 to 0.269, leading to a reduction of 5.6%. Compared to the influence of the Ni volume fraction, the influence of the LSM volume fraction can be almost negligible. This is because the cathode has lower material strength and smaller thickness compared to the anode. Changes in its mechanical properties have a less obvious effect on the stress distribution of other components. Therefore, the LSM volume fraction with a higher electrochemical efficiency can be selected for cathode manufacturing.

Both the anode and cathode are composite materials. The anode is sintered from a mixture of Ni/NiO and YSZ, while the cathode is sintered from a mixture of LSM and YSZ. The physical and mechanical parameters of different material components vary, so the proportion of different materials directly affects the material parameters of the composite materials. In engineering structural design, a failure probability of Pf ≤ 0.01 (1%) is typically adopted as a reference target for high-reliability design. However, the specific threshold must ultimately be determined based on a risk assessment of the application scenario, the statistical confidence of the material data, and the size effect of the component. It can be seen that SOFCs sealed with GC materials are prone to failure. As a result, many researchers are dedicated to improving the sealing reliability of SOFCs, such as developing SOFCs that operate at intermediate-to-low temperatures, creating novel sealing materials, and optimizing structural configurations.

It should be noted that in addition to material composition, the effect of porosity on mechanical properties is also critical. This paper neglected the effect of porosity on the CTE of the electrodes. Actually, porosity plays a critical role in determining the CTE, as pores do not contribute to thermal expansion. Consequently, a higher porosity typically results in a lower CTE [34,35]. This is the limitation of this paper, and we will improve it and study the influence of porosity on the CTE in the future. The mechanical properties of cermet electrodes are combined effects of the Ni and YSZ phases. Therefore, changes in composition naturally lead to changes in properties. However, mechanical properties such as elastic modulus, creep rate, and strength are also strongly influenced by porosity, not only by composition. Clarifying or incorporating the role of porosity would strengthen the interpretation of the creep behavior and failure probability. In our subsequent research, we will investigate the influence of porosity on creep lifetime.

## 4. Conclusions

The effects of the material composition of the anode and cathode on the creep damage and failure probability of SOFC after 50,000 h are investigated by the finite element method and the Weibull method, respectively. The following conclusions are achieved:

(1) The maximum principal stresses after creep, creep strain, and creep damage of SOFC gradually decrease, reaching a minimum at a volume fraction of 60%, with the Ni volume fraction decreasing. When the volume fraction increases from 60% to 70%, the maximum principal stress, creep strain, and creep damage of the SOFC increase again.

(2) The sealant is the most prone to failure and the most dangerous part. When the Ni volume fraction is 50% or 60%, the failure probabilities of the sealant are 0.72 and 0.012, respectively. When the Ni volume fraction is 30%, 40% and 70%, the failure probability of the sealant reaches one. The Ni volume fraction of 50–60% is appropriate to maintain the long-term operation of SOFC.

(3) The creep strain and creep damage of the other components are small, with very little change. The LSM volume fraction effect is not significant. The effect of the LSM cathode volume fraction is significantly weaker than that of the anode Ni content; therefore, it can be almost negligible. In conclusion, the LSM volume fraction with higher electrochemical efficiency can be selected for cathode manufacturing.

## Figures and Tables

**Table 1:** Material parameters of SOFC [26].

Component	Material	Thickness (µm)	Young’s Modulus (GPa)	Poisson’s Ratio	CTE (°C^−1^ × 10^−6^)
Anode	NiO-YSZ	600	108.0 (20 °C) 103.3 (800 °C)	0.3	11.7 (20 °C) 12.41 (800 °C)
Electrolyte	YSZ	10	200.0 (20 °C) 157.0 (800 °C)	0.31	7.6 (20 °C) 10.0 (800 °C)
Cathode	LSM	40	41.0 (20 °C) 48.0 (800 °C)	0.28	9.8 (20 °C) 11.4 (800 °C)
Sealant	glass-ceramic (GC)	100	66.0 (20 °C) 16.0 (800 °C)	0.28	11.1 (20 °C) 11.1 (800 °C)
Frame	Crofer 22apu	500	214.0 (20 °C) 44.0 (800 °C)	0.29	10.0 (20 °C) 11.9 (800 °C)

**Table 2:** Creep parameters and Weibull parameters [28,29].

Creep Parameters	Ni-YSZ	YSZ	LSM	GC	Crofer 22 APU
*B* (MPa^−n^h^−1^)	9.504 × 10^−8^	4.245 × 10^−11^	4.573 × 10^−9^	8.478 × 10^−9^	1.3752 × 10^−10^
*n*	1.7	1	1.7	5.943	6
*ε_f_*	0.02	0.02	0.02	0.01	0.6
*m*	17.8	8.6	3.7	6.0	
*V*_0_	0.578	1.21	0.35	1.0	
η	0.003	0.003	0.003	0.0013	

**Table 3:** Material properties of dense materials for each component of electrodes [19,26].

Material	Young’s Modulus (GPa)	Poisson Ratio	CTE (10^−6^ °C^−1^)	Density (10^3^ kgm^−3^)
Ni (RT)	200	0.313	14.2	8.9
Ni (800 °C)	171		16.9	
NiO (RT)	220	0.317	10.6	6.67
NiO (800 °C)	190		14.1	
YSZ (RT)	215	0.317	7.6	6.04
YSZ (800 °C)	185		10.5	
LSM	95	0.32	12.4	6.57

## Data Availability

The original contributions presented in the study are included in the article. Further inquiries can be directed to the corresponding author.
